# Role of Ran-regulated nuclear-cytoplasmic trafficking of pVHL in the regulation of microtubular stability-mediated HIF-1α in hypoxic cardiomyocytes

**DOI:** 10.1038/srep09193

**Published:** 2015-03-17

**Authors:** Xupin Jiang, Dongxia Zhang, Hengshu Zhang, Yuesheng Huang, Miao Teng

**Affiliations:** 1 Institute of Burn Research, State Key Laboratory of Trauma, Burns and Combined Injury, Southwest Hospital, The Third Military Medical University, Chongqing, China; 2 Department of Burn and Plastic Surgery, The First Affiliated Hospital of Chongqing Medical University, Chongqing, China

## Abstract

Our previous study suggested that microtubule network alteration affects the process of glycolysis in cardiomyocytes (CMs) via the regulation of hypoxia-inducible factor (HIF)-1α during the early stages of hypoxia. However, little is known regarding the underlying mechanisms of microtubule network alteration-induced changes of HIF-1α. The von Hippel–Lindau tumor suppressor protein (pVHL) has been shown to mediate the ubiquitination of HIF-1α in the nuclear compartment prior to HIF-1α exportation to the cytoplasm, and pVHL dynamic nuclear-cytoplasmic trafficking is indicated to be involved in the process of HIF-1α degradation. In this study, by administering different microtubule-stabilizing and -depolymerizing interventions, we demonstrated that microtubule stabilization promoted pVHL nuclear export and drove the translocation of pVHL to the cytoplasm, while microtubule disruption prevented pVHL nuclear export in hypoxic CMs. Moreover, the ratio between nuclear and cytoplasmic pVHL was associated with HIF-1α regulation. Importantly, microtubule network alteration also affected the subcellular localization of Ran, which was involved in the regulation of pVHL nuclear-cytoplasmic trafficking. The above results suggest that the subcellular translocation of pVHL plays an important role in microtubular structure alteration-induced HIF-1α regulation. Interestingly, Ran is involved in the process of pVHL nuclear-cytoplasmic trafficking following microtubule network alteration in hypoxic CMs.

Hypoxia is a common pathophysiological process for various ischemic diseases. Myocardial hypoxia is presented in patients with coronary artery disease, hypertensive heart disease, and cardiomyopathy^1^,^2^, as well as those with severe burns^3^,^4^. Cytoskeletal disruption has been recognized as a hallmark of the cellular response to hypoxia or ischemia, especially the changes of the microtubule network^5^,^6^,^7^. Our previous work has revealed that alterations of the microtubule network in hypoxic cardiomyocytes (CMs) may impact the process of glycolysis, and hypoxia-inducible factor (HIF-1α) is indicated to be the dominant mediator^8^. However, the exact mechanisms underlying microtubule alteration-associated regulation of HIF-1α in hypoxic CMs are still unknown.

HIF-1α is a transcriptional factor that has been suggested to modulate the cellular responses to hypoxia^9^ and to stimulate the processes of erythropoiesis, angiogenesis, and intracellular metabolism^10^. Under normoxic conditions, the von Hippel–Lindau tumor suppressor protein (pVHL) recognizes HIF-1α as a substrate for the enzymatic modification by prolyl hydroxylases, ubiquitination, and subsequent degradation by the 26S proteasome^11^. Conversely, in hypoxic cells, HIF-1α escapes from degradation as a result of inactivation of prolyl hydroxylases^12^,^13^,^14^. However, the exact effect of pVHL on HIF-1α expression in hypoxic CMs has not been investigated in previous studies. We found previously that microtubule network stabilization downregulates pVHL, which in turn leads to an increase of HIF-1α protein expression. Meanwhile, microtubule network breakdown also promotes pVHL-mediated HIF-1α degradation in hypoxic CMs^15^. Indeed, previous evidence has confirmed that pVHL mediates ubiquitination of HIF-1α in the nuclear compartment prior to HIF-1α exportation to the cytoplasm and that pVHL dynamic nuclear-cytoplasmic trafficking is required for HIF-1α degradation^16^. Moreover, our previous study also has revealed that the stabilization of microtubular structures in hypoxic CMs increases HIF-1α protein expression and promotes its accumulation in the nucleus^8^. Therefore, we hypothesized that microtubule alteration may regulate HIF-1α degradation via affecting the process of pVHL nuclear-cytoplasmic trafficking.

The transport of macromolecules between the nucleus and cytoplasm is an energy-dependent process. Substrates are translocated across the nuclear envelope via the nuclear pore complexes^17^. Ran is a member of the Ras family of small GTP-binding proteins, which were originally discovered as shuttling proteins with nuclear localization sequences^18^. Ran has been suggested to be involved in pVHL nuclear export in renal clear cell carcinoma^19^. Previous studies also have shown that GTPase may be implicated in cilia trafficking as well as the regulation of microtubule assembly dynamics and organization^20^,^21^,^22^, indicating the potential interaction between Ran and the microtubule network. Accordingly, these data also prompted us to investigate the function of Ran in the regulation of microtubule alteration-associated pVHL nuclear-cytoplasmic trafficking in hypoxic CMs. Therefore, in this study, we aimed to investigate the role of pVHL nuclear-cytoplasmic trafficking in the process of microtubule alteration-regulated HIF-1α degradation. Moreover, we evaluated whether Ran is involved in the process as a key regulator of pVHL nuclear-cytoplasmic trafficking.

In the present study, we found that microtubule stabilization promoted pVHL nuclear export, while microtubule disruption prevented pVHL nuclear export in hypoxic CMs. We also showed that pVHL nuclear-cytoplasmic trafficking plays an important role in subsequent HIF-1α regulation. Furthermore, microtubule network alteration also affected the subcellular localization of Ran, which was involved in the regulation of pVHL nuclear-cytoplasmic trafficking. Collectively, our findings suggest that Ran-mediated pVHL nuclear-cytoplasmic trafficking following microtubular network alteration is critical for HIF-1α regulation in hypoxic CMs. These results provide novel insights into mechanisms of HIF-1α regulation by microtubule alteration in hypoxic CMs.

## Results

### Microtubule stabilization prevents pVHL nuclear import in hypoxic CMs

**Figure 1:**
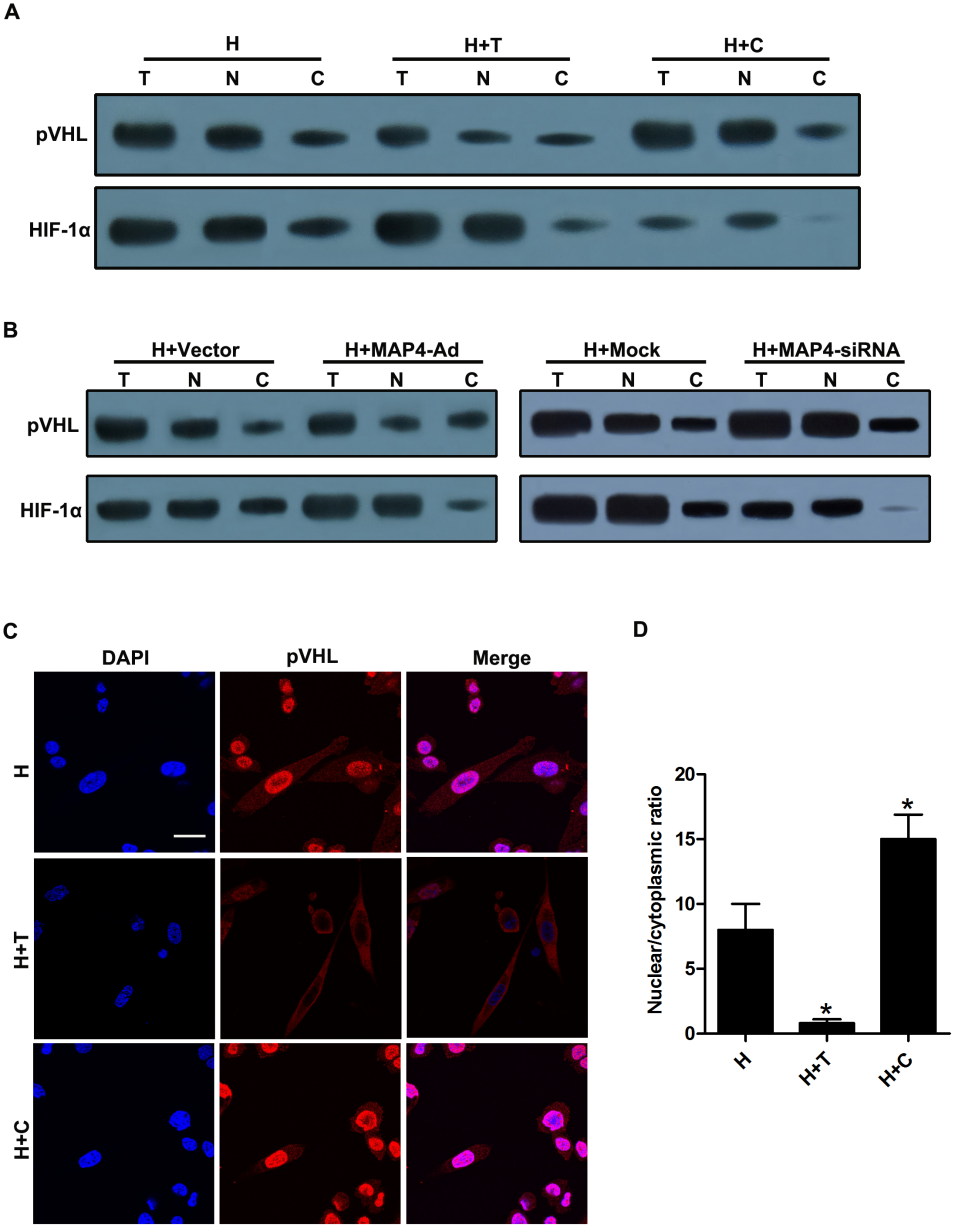
Microtubule disruption prevented pVHL nuclear export in hypoxic CMs. To observe the effect of microtubule interference on pVHL trafficking, CMs were treated with paclitaxel, colchicine, or recombinant adenovirus vectors to overexpress or silence MAP4 prior to hypoxia for 3 h. (A and B) Biochemical subcellular fractionation was performed with the digitonin system to obtain total (T), nuclear (N), and cytosolic (C) fractions, which were analyzed by western blot with anti-pVHL and anti-HIF-1α antibodies. (C) Hypoxic CMs pretreated with microtubule-interfering agents were stained with pVHL antibody (red). Nuclei were stained with DAPI (blue). Scale bar = 50 μm. (D) Nuclear/cytoplasmic ratios were calculated for 30 cells for each condition obtained from at least three independent experiments. Means with the standard deviations are shown. *, P < 0.05 versus the H group. CMs, cardiomyocytes; H, hypoxia; H + T, hypoxia and paclitaxel; H + C, hypoxia and colchicine; H + MAP4-Ad, hypoxia and MAP4 overexpression; H + MAP4-siRNA, hypoxia and MAP4 silencing.

To investigate whether the microtubule network alteration affects pVHL trafficking, we treated CMs for 3 h with paclitaxel and colchicine as well as vectors to increase or decrease MAP4 expression under hypoxic conditions. Subsequently, we assessed the subcellular localization of pVHL and HIF-1α by immunoblotting and immunofluorescence experiments. In hypoxic CMs, pVHL and HIF-1α mainly resided in the nuclear compartment. In fact, HIF-1α ubiquitination occurs in the nuclear compartment. When the microtubule network was restabilized with paclitaxel or after the administration of the vector MAP4-Ad, the pVHL protein level in the nuclear compartment as well as the ratio between the nuclear and cytosolic pVHL decreased. We also found that HIF-1α protein expression was upregulated and mainly located in the nucleus. However, pVHL accumulated almost exclusively in the nuclear compartment after the microtubule network was further disrupted using colchicine pretreatment or MAP4-siRNA vector administration. This result was associated with HIF-1α downregulation in the total and nuclear fractions (Fig. 1). Taken together, these results demonstrated that microtubule alteration regulates the subcellular localization of pVHL and the protein level of HIF-1α in hypoxic CMs.

### Microtubule alteration-mediated pVHL subcellular trafficking is involved in HIF-1α regulation

**Figure 2:**
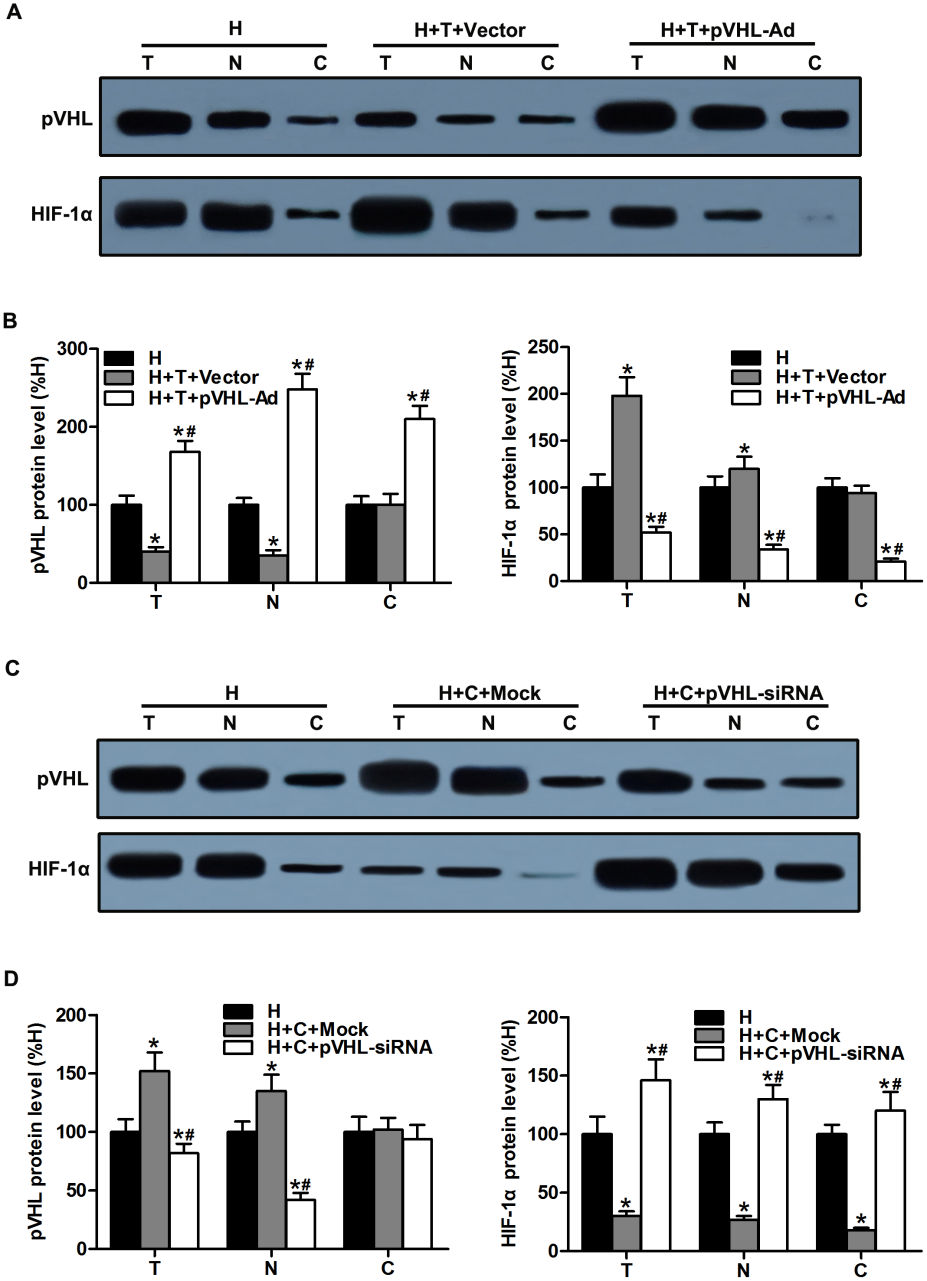
Microtubule alteration-mediated pVHL subcellular localization trafficking was involved in HIF-1α regulation. (A) CMs were transfected with recombinant adenovirus to overexpress pVHL (pVHL-Ad) before treatment with paclitaxel prior to hypoxia. Western blots are shown for pVHL and HIF-1α in the total (T), nuclear (N), and cytosolic (C) fractions in hypoxic CMs. (B) The graphs depict the mean ± SD (n = 3) of the relative integrated signal. (C) CMs were transfected with recombinant adenovirus to silence pVHL (pVHL-siRNA) before treatment with colchicine prior to hypoxia. Western blots are shown for pVHL and HIF-1α in the total (T), nuclear (N), and cytosolic (C) fractions in these hypoxic CMs. (D) The graphs depict the mean ± SD (n = 3) of the relative integrated signal. CMs, cardiomyocytes; H, hypoxia; H + T + Vector, hypoxia, paclitaxel, and the negative control; H + T + pVHL-Ad, hypoxia, paclitaxel, and pVHL overexpression; H + C + Mock, hypoxia and Mock transfection (as a negative control of pVHL-siRNA transfection); H + C + pVHL-siRNA, hypoxia, colchicine, and pVHL silencing; *, P < 0.05 versus the H group; ^#^, P < 0.05 versus the H + T + Vector or H + C + Mock group.

It has been well documented that microtubule network alteration results in changes of HIF-1α^8^,^23^ and HIF-1α ubiquitylation, which require that pVHL is present in the nucleus, and its degradation is a cytoplasmic event^24^. Therefore, we sought to determine whether the changes of HIF-1α protein expression depend on microtubule alteration-mediated pVHL subcellular trafficking. CMs were pretreated with paclitaxel or pVHL-siRNA adenovirus to knock down pVHL and were transfected with pVHL recombinant adenoviral vectors to induce the expression of pVHL. In paclitaxel-treated cells, pVHL in the nuclear compartment was significantly increased and the ratio between the nuclear and cytosolic pVHL also increased after pVHL-Ad administration, which correlated with HIF-1α downregulation (Fig. 2A–B). In contrast, pVHL in the nuclear compartment was reduced and the ratio of nuclear/cytosolic pVHL decreased after pVHL silencing, resulting in the upregulation of HIF-1α proteins (Fig. 2C–D). These results suggested that pVHL subcellular localization plays an important role in HIF-1α regulation following microtubule interference in hypoxic CMs.

### Microtubule network affects Ran subcellular localization in hypoxic CMs

**Figure 3:**
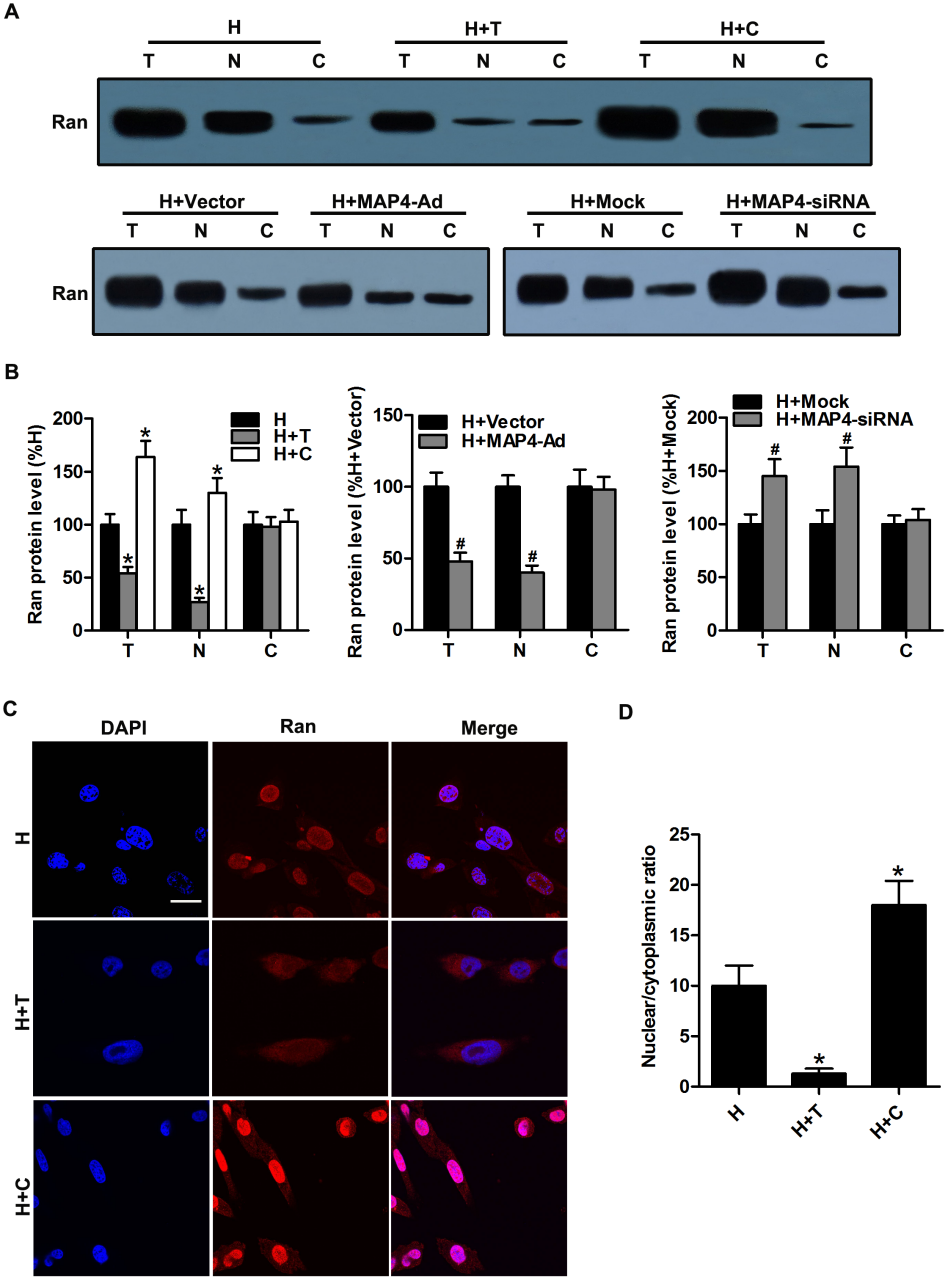
Microtubule network affected Ran subcellular localization in hypoxic CMs. (A) Western blots are shown for Ran in the total (T), nuclear (N), and cytosolic (C) fractions in hypoxic CMs with microtubule interference. (B) The graphs depict the mean ± SD (n = 3) of the relative integrated signal. (C) Hypoxic CMs pretreated with microtubule-interfering agents were stained with Ran antibody (red). Nuclei were stained with DAPI (blue). Scale bar = 50 μm. (D) Nuclear/cytoplasmic ratios were calculated for 30 cells for each condition obtained from at least three independent experiments. Mean averages with the standard deviations are shown. CMs, cardiomyocytes; *, P < 0.05 versus the H group; ^#^, P < 0.05 versus the H + Vector or H + Mock group.

Since Ran is considered as a key component of the nuclear/cytoplasmic transport machinery^17^,^25^ and is implicated in microtubule reorganization^26^,^27^, we detected the protein levels of Ran in the nuclear compartment and the cytosolic fraction after microtubule alteration to assess whether microtubule changes affect the subcellular localization of Ran. Our results showed that the Ran protein expression in the nuclear compartment was downregulated and that the ratio between nuclear and cytosolic Ran decreased after restabilization of the microtubule network in hypoxic CMs (H + T and H + MAP4-Ad groups). Microtubule disruption induced Ran protein localization in the nuclear compartment and therefore increased the ratio between nuclear and cytosolic Ran (H + C and H + MAP4-siRNA groups) (Fig. 3A–B). The distribution of Ran in cells, detected by fluorescence microscopy, was also consistent with these findings (Fig. 3C–D). These results further confirmed that changes of the microtubule network regulate Ran subcellular localization in hypoxic CMs.

### Ran is required for microtubule alteration-mediated pVHL nuclear export

**Figure 4:**
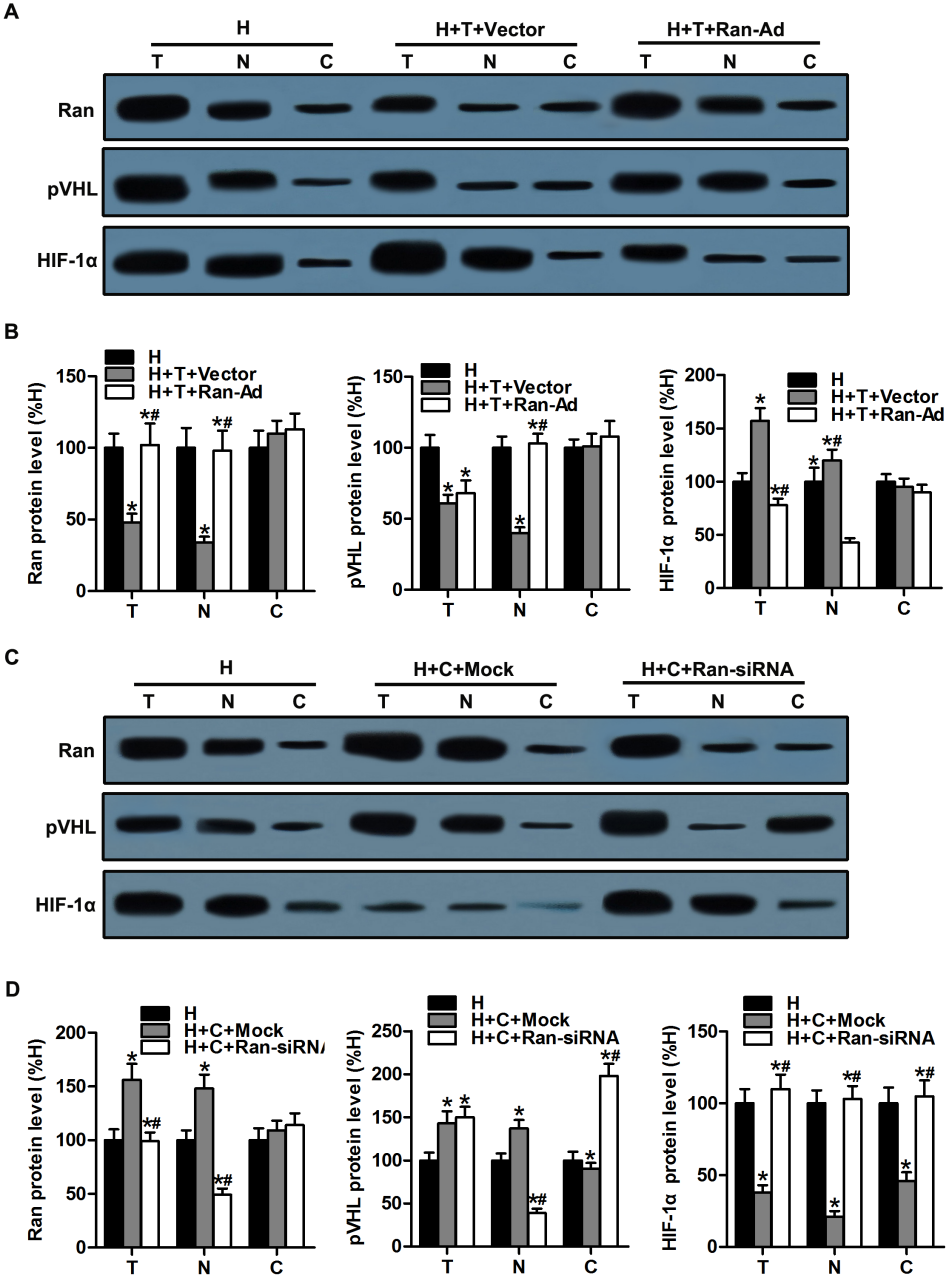
Ran was required for microtubule alteration-mediated pVHL nuclear export. (A) CMs were transfected with recombinant adenovirus to overexpress Ran (Ran-Ad) before treatment with paclitaxel prior to hypoxia. Western blots are shown for Ran, pVHL, and HIF-1α in the total (T), nuclear (N), and cytosolic (C) fractions in hypoxic CMs. (B) The graphs depict the mean ± SD (n = 3) of the relative integrated signal. (C) CMs were transfected with recombinant adenovirus to silence Ran (Ran-siRNA) before treatment with colchicine prior to hypoxia. Western blots are shown for Ran, pVHL, and HIF-1α in the total (T), nuclear (N), and cytosolic (C) fractions in these hypoxic CMs. (D) The graphs depict the mean ± SD (n = 3) of the relative integrated signal. CMs, cardiomyocytes; H, hypoxia; H + C + Mock, hypoxia and Mock transfection (as a negative control of Ran-Ad); H + C + Ran-siRNA, hypoxia, colchicine, and Ran silencing; H + T + Vector, hypoxia, paclitaxel, and the negative control of Ran overexpression; H + T + Ran-Ad, hypoxia, paclitaxel, and Ran overexpression; *, P < 0.05 versus the H group; ^#^, P < 0.05 versus the H + T + Vector or H + C + Mock group.

To investigate whether microtubule alteration-regulated pVHL nuclear export depends on the trafficking of Ran, we treated CMs with Ran-Ad or Ran-siRNA vector to up- or downregulate Ran expression before the CMs were treated with microtubule-interfering agents (paclitaxel or colchicine). Compared with the H + T + Vector group, the upregulated Ran was mainly located in the nuclear compartment and the ratio between nuclear and cytosolic Ran increased significantly, which resulted in the upregulation of pVHL protein expression in the nuclear compartment and the subsequent degradation of HIF-1α (Fig. 4A–B). Meanwhile, in CMs pretreated with colchicine, pVHL protein expression in the nuclear compartment was downregulated and the ratio between the nuclear and cytosolic Ran was decreased following Ran silencing, which induced HIF-1α expression in hypoxic CMs (Fig. 4C–4D). These results indicated that Ran plays important roles in both the process of pVHL subcellular trafficking and HIF-1α regulation mediated by microtubule alterations in hypoxic CMs.

### Ran physically interacts with α-tubulin and pVHL

**Figure 5:**
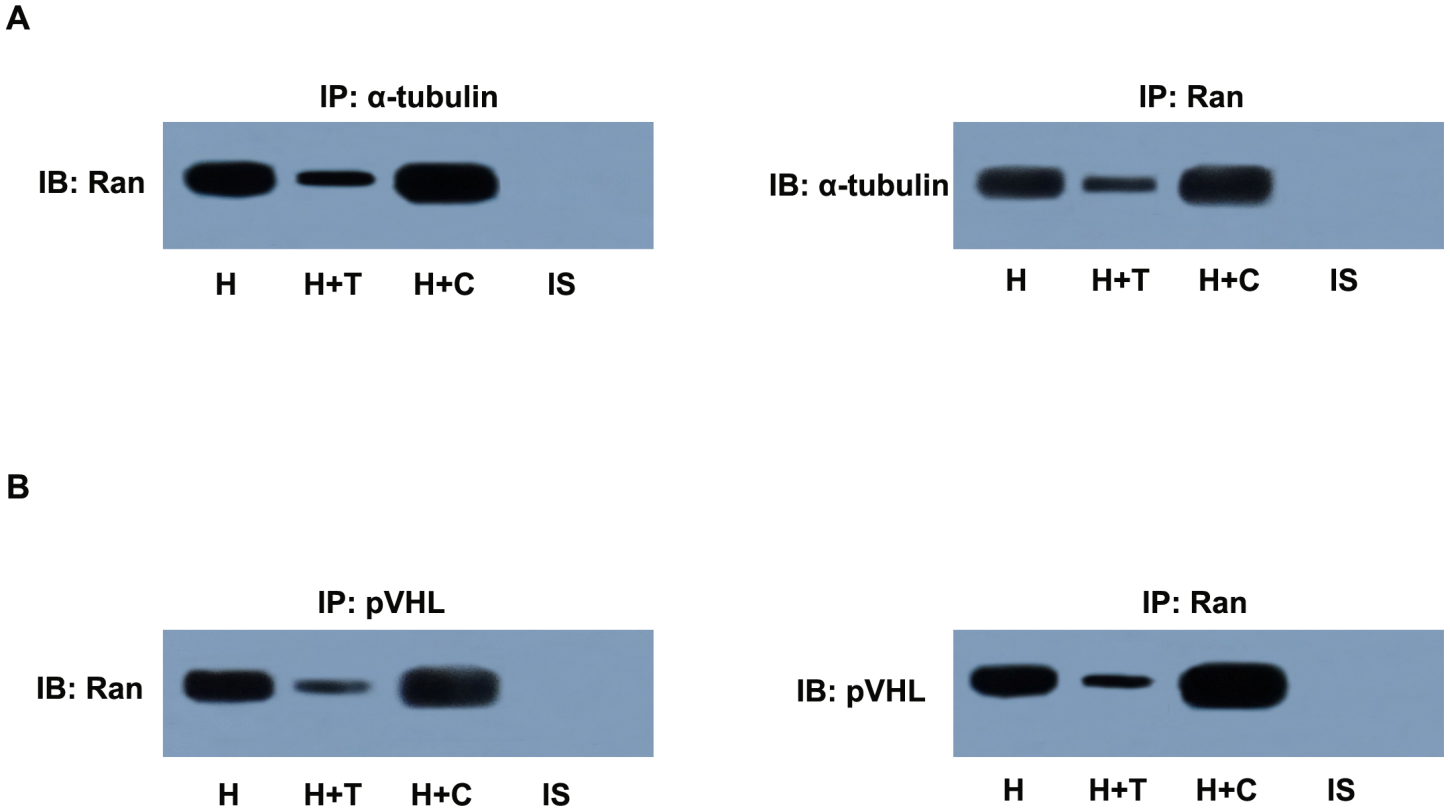
Ran physically interacted with α-tubulin and pVHL. Hypoxic CMs were pretreated with paclitaxel (H + T) or colchicine (H + C). (A) Cell extracts were immunoprecipitated by anti-Ran or anti-α-tubulin antibody and then immunoblotted with anti-α-tubulin or anti-Ran antibody. (B) Cell extracts were immunoprecipitated by anti-Ran or anti-pVHL antibody and then immunoblotted with anti-pVHL or anti-Ran antibody. CMs, cardiomyocytes; IP, immunoprecipitation; IB, immunoblotting; IS, isotype.

Microtubules form networks in the cytoplasm and play pivotal roles in cell polarity as well as the intracellular transportation of various biomolecules^27^. Microtubule-interfering agents (paclitaxel or colchicine) function to change the microtubule network via interaction with α/β-tubulin heterodimers^28^. The microtubule network alteration induced by paclitaxel or colchicine may affect Ran trafficking by forming a complex with α-tubulin. In addition, since Ran is involved in pVHL nuclear/cytoplasmic transport, it may bind to pVHL. Indeed, in CMs pretreated with paclitaxel or colchicine, our results showed that Ran was co-immunoprecipitated by α-tubulin or pVHL. Conversely, α-tubulin or pVHL could be co-immunoprecipitated by Ran under hypoxic conditions (Fig. 5). These results suggested that Ran physically interacts with α-tubulin and pVHL and can be regulated by microtubule alteration in hypoxic CMs.

## Discussion

The microtubule network is a major component of the cytoskeleton and is crucial for the maintenance of normal cellular physiology. Our previous results revealed that microtubule interference affects glycolysis during early hypoxia via regulation of HIF-1α in hypoxic CMs. HIF-1α degradation requires its ubiquitination to be mediated by pVHL in the nuclear compartment and its subsequent exportation to the cytoplasm^16^. As we have shown that microtubule network alteration changes the HIF-1α protein level via regulation of pVHL expression^15^, we wondered whether microtubule regulated-pVHL trafficking to the nucleus, a required step for HIF-1α degradation, is also involved in microtubule-dependent regulation of HIF-1α protein in CMs during the early stages of hypoxia. In this study, we demonstrated that microtubule alteration affected pVHL nuclear-cytoplasmic trafficking, which was involved in HIF-1α regulation. Moreover, the subcellular localization of Ran mediated by microtubule alterations participated in the regulation of pVHL nuclear-cytoplasmic trafficking and HIF-1α degradation. This work, together with our previous data^15^, demonstrates that microtubule alteration may regulate HIF-1α protein levels in hypoxic CMs by two distinct mechanisms. Microtubule alteration could not only regulate the pVHL protein levels, but it also affected pVHL nuclear-cytoplasmic trafficking, which subsequently resulted in HIF-1α changes.

It is now understood that microtubules are critical for various cellular processes such as cell movement, vesicle and organelle transport, and mitosis. It is also clear that microtubules are essential for the intracellular trafficking of viruses^29^, protein complexes, and other transcriptional factors^30^,^31^,^32^. Accumulating evidence has revealed that microtubule network alteration may lead to changes in HIF-1α protein levels^23^,^33^, trafficking, and activity^34^. Indeed, our previous study revealed that microtubular structural changes regulate HIF-1α protein levels but not mRNA levels in CMs during the early stages of hypoxia. Cytoplasmic accumulation of HIF-1α is very transient and difficult to detect due to its quick degradation and very short half-life upon reoxygenation^35^. Moreover, HIF-1α localization is indicated to be restricted to the nuclear compartment in hypoxic cells. Stabilizing microtubular structures in hypoxic CMs increases the HIF-1α protein level and promotes its accumulation in the nuclei, while microtubular structure breakdown decreases its protein level and accumulation^8^. pVHL, a component of the E3 ubiquitin protein-ligase complex, functions to target HIF-1α for degradation via the ubiquitin proteasome pathway^36^,^37^. pVHL is also known to be associated with at least four other molecules, including elongin B, elongin C, Cullin-2, and Rbx1^38^. In addition, pVHL assembles with HIF-1α solely in the nuclear compartment, and endogenous HIF-1α can only be detected in the nuclear compartment. Furthermore, at least a portion of HIF-1α is immediately imported into the nucleus and is subjected to nuclear ubiquitination and subsequent nuclear export for cytoplasmic degradation^16^. In the present study, we demonstrated that microtubule stability decreased the pVHL protein level in the nuclear compartment of hypoxic CMs and thereby induced the HIF-1α protein expression level, while pVHL and HIF-1α accumulated almost exclusively in the nuclear compartment following further microtubule network disruption (Fig. 1). Therefore, these results indicated that microtubule alterations may be a key regulator for pVHL nuclear-cytoplasmic trafficking and HIF-1α degradation in hypoxic CMs.

Indeed, the ability of pVHL to shuttle between the nuclear and cytoplasmic compartments has been implicated in the ubiquitination and degradation of HIF-1α in previous studies^16^,^39^. pVHL is indicated to be engaged in a constitutive shuttle between the nucleus and the cytoplasm^40^. Increasing evidence has shown that pVHL is also associated with microtubule dynamics^41^,^42^,^43^ and microtubule network alteration in hypoxic cells^15^,^44^. Moreover, our results revealed that the HIF-1α protein level and the ratio between the nuclear and cytosolic HIF-1α in hypoxic CMs were reduced after upregulation of pVHL in the nuclear compartment and following microtubule stabilization. Conversely, when pVHL in the nuclear compartment was downregulated and its ratio between the nuclear and cytosolic compartments was reduced, the HIF-1α protein level was significantly upregulated in hypoxic CMs following microtubule disruption (Fig. 2). Accordingly, these results further confirmed that pVHL subcellular localization plays an important role in HIF-1α regulation after microtubule network alteration in hypoxic CMs.

Next, we pursued our investigation to identify the potential mechanisms responsible for pVHL exportation from the nucleus. It has been demonstrated in previous studies that at least four independent factors are required for efficient nuclear export of pVHL: an ATP hydrolysis step, the presence of GTP, the synthesis of polyadenylated RNA, and the small GTPase Ran^19^. In addition, ATP hydrolysis has been shown to be necessary for nuclear export of other exportable substrates^45^. Interestingly, we found that microtubule alteration led to a change of Ran subcellular localization in hypoxic CMs (Fig. 3). Ran has been suggested to influence many cellular processes, including nuclear import and export of proteins, the construction and/or integrity of the nuclear envelope, and spindle formation during mitosis^27^. In interphase, GDP-bound Ran resides in the cytoplasm. Whenever in the nucleus, GDP on Ran is exchanged for GTP by the action of chromatin-bound RanGEF, the guanine nucleotide exchange factor of Ran, yielding Ran-GTP. The nuclear envelope serves as a nucleo-cytoplasmic barrier for Ran-GTP^46^. Furthermore, increasing data indicate that Ran may be involved in the regulation of microtubule organization^47^,^48^. Our results revealed that an increased ratio between nuclear and cytosolic Ran results in upregulation of the pVHL protein level in the nuclear compartment, which subsequently regulates HIF-1α degradation in hypoxic CMs (Fig. 3–4). Besides, Ran co-immunoprecipitated with α-tubulin and pVHL in hypoxic CMs, which suggests a possible physical interaction of microtubule alteration-induced pVHL nuclear-cytoplasmic trafficking through Ran. These findings suggested that Ran is involved in the changes of pVHL subcellular localization induced by microtubule network alteration, but it remains unclear whether Ran interacts with pVHL directly or indirectly. Future studies are expected to reveal the details of this process.

Microtubules form networks in the cytoplasm and play key roles in cell polarity and intracellular transport of various biomolecules^49^. Previous studies have suggested a potential role of Ran in microtubule dynamics^48^. It is well known that pVHL localizes predominantly to the cytoplasmic compartment, but engages in a dynamic nuclear-cytoplasmic shuttle^39^,^50^, and that pVHL mediates ubiquitination of HIF-1α in the nuclear compartment prior to HIF-1α exportation to the cytoplasm^16^. However, little is known about how pVHL is involved in the dynamic process. Our results showed that microtubular alteration-mediated Ran regulation plays an important role in the process of pVHL nuclear-cytoplasmic shuttling and HIF-1α degradation. The data provide a deeper insight into the mechanism of pVHL shuttling and HIF-1α regulation.

**Figure 6:**
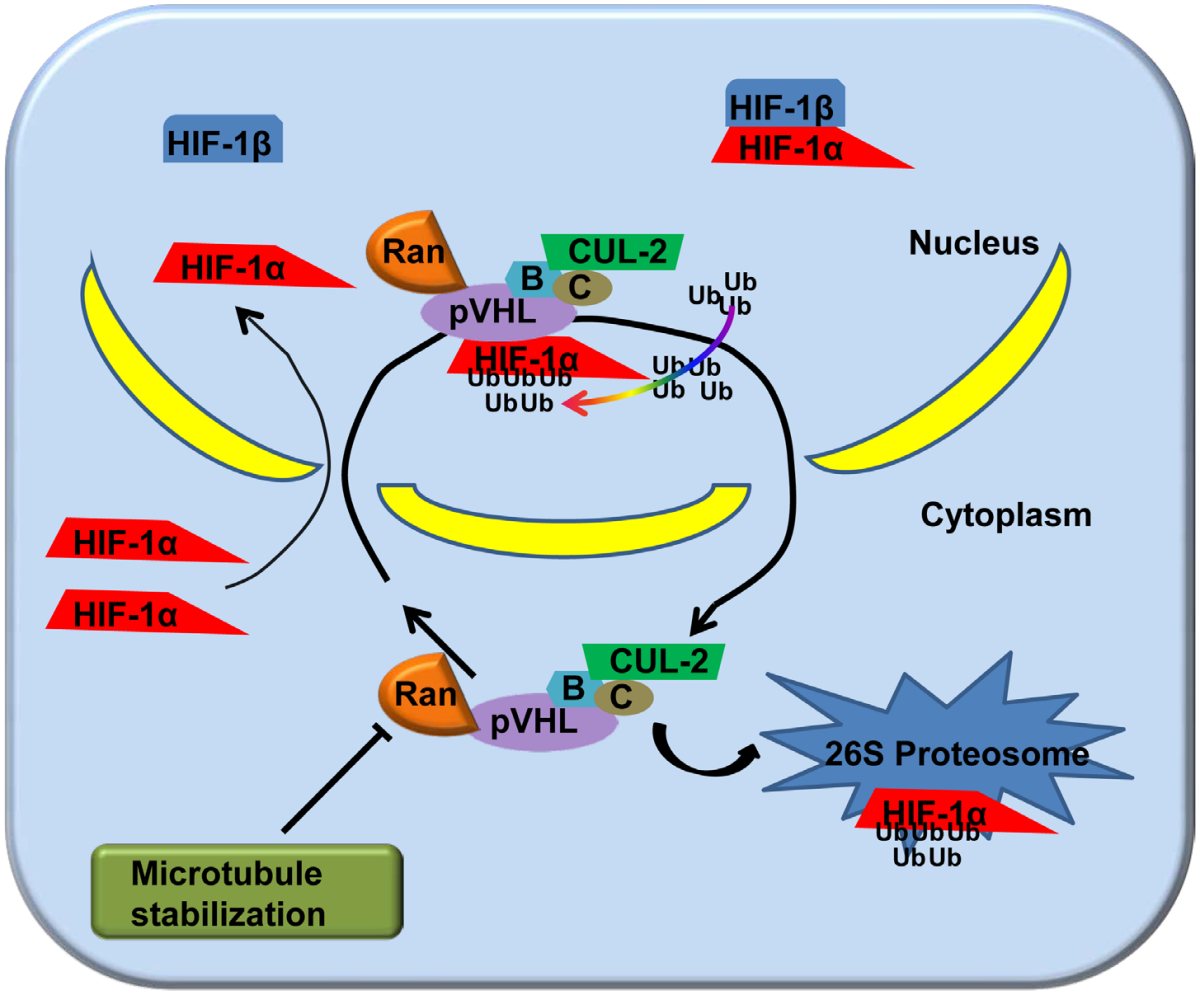
Schematic images showing the potential interactions among microtubule alteration, Ran/pVHL nuclear-cytoplasmic trafficking, and HIF-1α degradation in hypoxic CMs. In hypoxic CMs, the HIF-1α protein level has a dynamic balance. On the one hand, HIF-1α is immediately imported into the nucleus, where it binds to HIF-1β to form the transcription factor HIF, which transactivates hypoxia-inducible genes. On the other hand, HIF-1α incurs a posttranslational modification in the nuclear compartment. Nuclear HIF-1α binds to pVHL and undergoes Cullin-2-mediated ubiquitination (Ub) prior to exportation to the cytoplasm for 26S proteasomal degradation. pVHL engages in a constitutive nuclear-cytoplasmic shuttle, which is mediated by Ran. Nuclear HIF-1α embarks on the pVHL/BC/Cul-2 complex, depending upon its ability to assemble with nuclear pVHL. Microtubule stabilization inhibits Ran-mediated pVHL nuclear accumulation, which in turn leads to HIF-1α protein upregulation. Conversely, microtubule network breakdown upregulated Ran-mediated pVHL nuclear accumulation and promoted HIF-1α degradation in hypoxic CMs. CMs, cardiomyocytes.

Taken together, we observed that microtubule network stabilization downregulated Ran-mediated pVHL nuclear accumulation, which in turn led to the upregulation of HIF-1α protein (Fig. 6). HIF-1α, a key regulator of the molecular hypoxic response, can mediate a wide range of physiological and cellular processes that are necessary for cells to adapt to hypoxic conditions^51^. The upregulation of HIF-1α in CMs has been considered to be protective against myocardial ischemia *in vivo*^52^,^53^. In addition, the microtubule stabilizer taxol has been reported to prevent ischemic ventricular arrhythmias in rats^54^. These results may reflect the fact that microtubule network stabilization may be related to HIF-1α regulation under hypoxic conditions. Our findings may provide novel therapeutic targets for regulating HIF-1α and attenuating hypoxia-related myocardial injury.

## Methods

### Cell culture

Neonatal Sprague-Dawley rats (1–2 days old) were provided by the Animal Center of the Third Military Medical University. The animal-based investigations were designed in accordance with the Guide for the Care and Use of Laboratory Animals published by the National Institutes of Health (NIH Pub. No. 85–23, revised 1996), and the entire project was reviewed and approved by the Animal Experiment Ethics Committee of the Third Military Medical University (Approval Number SYXK-CQ-20070002). Rat ventricular myocardia were harvested and digested with trypsin and then cultured according to the protocols published previously^55^. Neonatal rat ventricular CMs were cultured in Dulbecco's modified Eagle's medium-F12 (Hyclone, USA) supplemented with 5-bromodeoxyuridine (31 mg/L), 10% heat-inactivated fetal bovine serum (Gibco, USA), penicillin G (100 U/mL), and streptomycin (100 mg/mL).

### Hypoxia treatment and microtubule interference

Hypoxic conditions of 1% O_2_, 5% CO_2_, and 94% N_2_ were created by a continuous flow of nitrogen using a Forma Series II Water Jacket CO_2_ incubator (model 3131; Thermo Scientific, USA). The recombinant adenovirus vectors were used to infect CMs before hypoxia treatment. Microtubule stabilization was achieved by treating the CMs with 10 mM paclitaxel (Sigma, USA) or infecting them with the MAP4 overexpression vector (MAP4-Ad) for 8 h prior to hypoxia treatment. Microtubule depolymerization was achieved by treating the CMs with 10 mM colchicine (Sigma, USA) or infecting them with the MAP4 silencing vector (MAP4-siRNA) for 8 h.

### Recombinant adenovirus vector for overexpression or silencing of MAP4, pVHL, or Ran expression

The recombinant adenovirus vectors for overexpression of MAP4, pVHL, or Ran (MAP4-Ad, pVHL-Ad, or Ran-Ad); the negative control adenovirus vector (Vector); the vectors for silencing of MAP4, pVHL, or Ran expression (MAP4-siRNA, pVHL-siRNA, or Ran-siRNA); and the negative vector containing nonspecific shRNA (Mock) were purchased from Shanghai GeneChem, Co. Ltd. (Shanghai, China). CMs were infected with these vectors at a multiplicity of 10 for 48 h before subsequent analyses.

### Subcellular fractionation

Subcellular fractions were extracted according to protocols described previously^16^. Briefly, cells were grown to confluence and treated as desired, then washed in cold phosphate-buffered saline (PBS), and scraped into transport buffer containing 20 mM HEPES (pH 7.3), 110 mM potassium acetate, 5 mM sodium acetate, 2 mM magnesium acetate, 1 mM EGTA, 2 mM dithiothreitol, and a protease inhibitor cocktail. Cells were left on ice for 2 min to equilibrate in the buffer before the addition of 50 g of digitonin/mL. Permeabilization was performed for 5–7 min on ice with gentle shaking and was monitored by fluorescence microscopy with Hoechst stain 33258, which only stained the nuclei of permeabilized cells. The permeabilization reaction was stopped when more than 99% of the cells displayed stained nuclei. The permeabilized cells were then centrifuged at 800 *g* to separate the nuclear fraction (pellet) from the cytosolic fraction (supernatant). The nuclei were washed in transport buffer containing the protease inhibitor cocktail, and the cytosolic fraction was centrifuged again to eliminate the remaining nuclei. The total fraction was prepared with the same amount of cells as that taken for fractionation. Cells of the total fraction and nuclear-cytosolic fractions were resuspended in an equal volume of lysis buffer or sodium dodecyl sulfate (SDS) loading buffer. An equal volume of each fraction was subjected to SDS–polyacrylamide gel electrophoresis (PAGE) analysis and western blot.

### Western blot analysis

The subcellular fractions of CMs in the different groups were extracted as described above. Cells of the total fraction, nuclear fraction, and cytosolic fraction were resuspended in an equal volume of SDS loading buffer. An equal volume of each fraction was subjected to separation on a 10% SDS–PAGE gel and transferred electrophoretically to polyvinylidene difluoride membranes. The blots were probed using the following primary antibodies: anti-pVHL (1:1000; Cell Signaling, USA), anti-HIF-1α (1:1000; Cell Signaling, USA), and anti-Ran (1:1000; Millipore, USA). The results were analyzed with the ChemiDoc imaging system (Bio-Rad, USA).

### Immunoprecipitation

Cells growing on a 60-mm dish were lysed in 300 mL of RIPA buffer containing 2 mM phenylmethylsulfonyl fluoride and a protease inhibitor cocktail. The anti-Ran antibody with anti-α-tubulin or anti-pVHL was incubated with 150 mL of cell lysate for 6 h at 4°C, and then the complexes were precipitated with protein A/G-Sepharose (Santa Cruz Biotechnology) overnight at 4°C. The precipitates were washed three times with PBS at 4°C and probed with anti-α-tubulin or anti-pVHL and anti-Ran antibodies using the western blotting technique described above.

### Immunofluorescence and image processing

CMs were pretreated with paclitaxel or colchicine prior to hypoxia treatment. Cells were then fixed in 4% paraformaldehyde for 20 min, permeabilized with 0.1% Triton X-100 in PBS for 20 min, and then blocked in 10% goat serum for 1 h. To observe the expression and subcellular localization of pVHL or Ran, the primary antibody mouse anti-pVHL (1:100; Cell Signaling, USA) or mouse anti-Ran (1:1000; Millipore, USA) was added to the cells, and the cells were incubated at 4°C overnight. The Cy3-conjugated goat anti-mouse secondary antibody (1:1000; Sigma-Aldrich, USA) was used. The nuclei were stained by 4, 6-diamidino-2-phenylindole (DAPI; 0.5 mg/mL; Biotium, China). Cells were imaged with confocal microscopy (TCS-NT; Leica, Wetzlar, Germany). Using the NIH Image J imaging software (http://rsb.info.nih.gov/ij/), the nuclear signal was obtained by measuring the pixel intensity of the nucleus subtracted by the mean background and then multiplying this value by the area of the nucleus. The cytoplasmic signal was obtained by subtracting the nuclear signal from the total signal. Finally, the nuclear-cytoplasmic ratio corresponded to the nuclear signal divided by the cytoplasmic signal.

### Statistical analysis

Normal distribution and homoscedasticity tests of the data were confirmed, and one-way analysis of variance followed by post-hoc tests were performed with SPSS 13.0 software. The results are presented as mean ± standard deviation (SD), and statistical significance was set at P < 0.05.

## Author Contributions

X.J. and M.T. developed initial concept. X.J., D.Z., H.Z., Y.H. and M.T. designed experiments. X.J., D.Z. and M.T. performed experiments and analysed data. X.J. wrote the manuscript. H.Z., Y.H. and M.T. supervised the study. All authors discussed the results and commented on the manuscript.

## Supplementary Material

Supplementary Information supplementary information
