# Supplementary material for: Role of Ran-regulated nuclear-cytoplasmic trafficking of pVHL in the regulation of microtubular stability-mediated HIF-1α in hypoxic cardiomyocytes
Source: Sci Rep. 2015 Mar 17;5:9193. doi: 10.1038/srep09193 (PMC4361876; doi:10.1038/srep09193)

Role of Ran-regulated nuclear-cytoplasmic trafficking of pVHL in the regulation of microtubular stability-mediated HIF-1α in hypoxic cardiomyocytes

Xupin Jiang[1,2], Dongxia Zhang[1], Hengshu Zhang[2], Yuesheng Huang[1]& Miao Teng[2]

[1] Institute of Burn Research, State Key Laboratory of Trauma, Burns and Combined Injury, Southwest Hospital, The Third Military Medical University, Chongqing, China,
[2] Department of Burn and Plastic Surgery, The First Affiliated Hospital of Chongqing Medical University, Chongqing, China

Correspondence and requests for materials should be addressed to M.T. (xpjiang1987@foxmail.com).

Department of Burn and Plastic Surgery, The First Affiliated Hospital of Chongqing Medical University, Chongqing, China.

Supporting Information

Supplemental Figure Legends

Figure S1. Assessment of the quality of subcellular fractionation using nuclear and cytosolic marker proteins. Western blot analysis of the protein expression of histone and α-tubulin in subcellular fractions of hypoxic CMs, which were pretreated with paclitaxel (H+T) or colchicine (H+C). Histone and α-tubulin were used as loading controls and markers for nuclear (N) and cytosolic (C) fractions.

Figure S2.Ran is required for microtubule alteration-mediated pVHL nuclear export. (A) Hypoxic CMs were pretreated with paclitaxel (H+T) or colchicine (H+C). Nuclear fractions were immunoprecipitated by anti-Ran or anti-pVHL antibody and then immunoblotted with anti-pVHL or anti-Ran antibody. Histone was used as a control for equal protein input loading. The western blots are representative examples of three experiments. (B) Densitometric quantification of the bands of three western blots as shown in A. Homogenates without treatment (H) were set as 100%. IP, immunoprecipitation; IB, immunoblotting; IS, isotype. (C) CMs were transfected with recombinant adenovirus to overexpress Ran (Ran-Ad) before treatment with paclitaxel, prior to hypoxia. The cells were immunostained with pVHL antibody (red). Nuclei were stained with DAPI (blue). Scale bar = 50 μm. (D) Nuclear/cytoplasmic ratios of pVHL were calculated for 30 cells for each condition obtained from at least three independent experiments. Mean averages with the SD are shown. *, $P < 0.05$ versus the H group; $^{\#}$,$P < 0.05$ versus the H+T+Vector group.

Figure S1

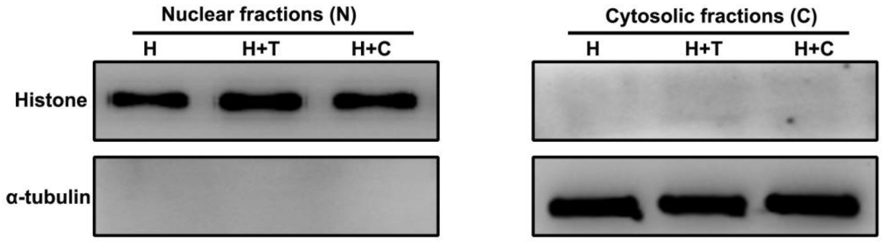

Figure S2

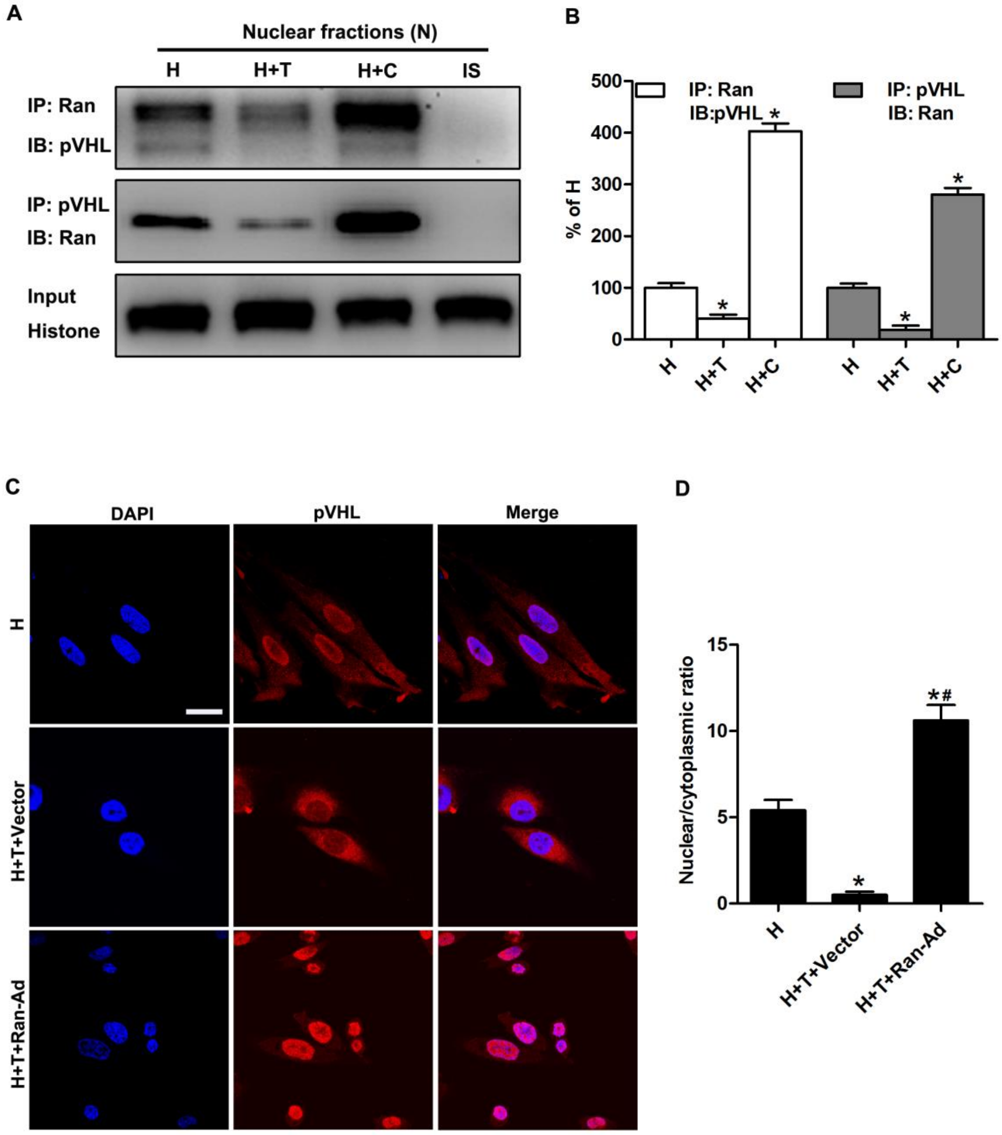

Supplement: Supplementary Information [file srep09193-s1.pdf]
